# Combining with lab-on-chip technology and multi-organ fusion strategy to estimate post-mortem interval of rat

**DOI:** 10.3389/fmed.2022.1083474

**Published:** 2023-01-10

**Authors:** Qiu-xiang Du, Shuai Zhang, Fei-hao Long, Xiao-jun Lu, Liang Wang, Jie Cao, Qian-qian Jin, Kang Ren, Ji Zhang, Ping Huang, Jun-hong Sun

**Affiliations:** ^1^Shanghai Key Laboratory of Forensic Medicine, Academy of Forensic Science, Shanghai, China; ^2^School of Forensic Medicine, Shanxi Medical University, Jinzhong, Shanxi, China; ^3^Criminal Investigation Detachment, Baotou Public Security Bureau, Baotou, Inner Mongolia, China; ^4^National Center for Liver Cancer, Second Military Medical University, Shanghai, China

**Keywords:** forensic pathology, machine learning, multi-organ fusion, lab-on-chip, post-mortem interval

## Abstract

**Background:**

The estimation of post-mortem interval (PMI) is one of the most important problems in forensic pathology all the time. Although many classical methods can be used to estimate time since death, accurate and rapid estimation of PMI is still a difficult task in forensic practice, so the estimation of PMI requires a faster, more accurate, and more convenient method.

**Materials and methods:**

In this study, an experimental method, lab-on-chip, is used to analyze the characterizations of polypeptide fragments of the lung, liver, kidney, and skeletal muscle of rats at defined time points after death (0, 1, 2, 3, 5, 7, 9, 12, 15, 18, 21, 24, 27, and 30 days). Then, machine learning algorithms (base model: LR, SVM, RF, GBDT, and MLPC; ensemble model: stacking, soft voting, and soft-weighted voting) are applied to predict PMI with single organ. Multi-organ fusion strategy is designed to predict PMI based on multiple organs. Then, the ensemble pruning algorithm determines the best combination of multi-organ.

**Results:**

The kidney is the best single organ for predicting the time of death, and its internal and external accuracy is 0.808 and 0.714, respectively. Multi-organ fusion strategy dramatically improves the performance of PMI estimation, and its internal and external accuracy is 0.962 and 0.893, respectively. Finally, the best organ combination determined by the ensemble pruning algorithm is all organs, such as lung, liver, kidney, and skeletal muscle.

**Conclusion:**

Lab-on-chip is feasible to detect polypeptide fragments and multi-organ fusion is more accurate than single organ for PMI estimation.

## 1. Introduction

Post-mortem interval (PMI), also called time since death, is the elapsed time between the death of an organism and the initiation of an official investigation (1). It is very important for the investigation of death in civil and criminal cases to accurately infer the time of death, such as civil investigation of life insurance fraud, identifying the victim and suspect, and accepting or rejecting the suspect’s alibi (2). Traditional inference methods of PMI are usually based on corpse temperature (3) and early corpse phenomena such as livor mortis (4), rigor mortis (5), and post-mortem turbidity of cornea (6); it is difficult to precisely confirm the time since death, because these methods are rough, subjective, and empirical, as well as are greatly affected by environmental factors (7).

With the development of biomolecular technology, detection methods based on nucleic acid (1, 8, 9), metabolites (10, 11), and microorganisms (2, 12, 13) have been widely used in the past few decades. Some studies suggested that the genes, such as GAPDH2, ACTB2, 18S rRNA, miR-1, and miR-133a, are suitable indicators for estimating PMI (14–16). The level of the metabolite, which was detected by nuclear magnetism, mass spectrometry, and spectrograph, also provided a new direction for PMI inference at the tissues level (17–20). A further investigation into microorganisms of human and animal remains to study microbial community succession after death (21–24). In addition, with the development of imaging technology, post-mortem computed tomography (25), microCT (26), and visible and thermal 3D imaging (27) have also been used to infer the time since death. These technologies provide valuable ideas and methods for PMI estimation in forensic practice.

Protein is one of the biological macromolecules, an essential component of the organism, and participates in every cellular process. In recent years, proteins, in particular, have been evaluated for their potential to aid PMI delimitation. Sodium dodecyl sulfate–polyacrylamide gel electrophoresis (SDS-PAGE)/western blotting (28, 29), immunohistochemistry (30, 31), and mass spectrometry (32, 33) were widely used to estimate the time since death. Although these approaches have shown some success and promise, there are certain limitations with these existing approaches, e.g., tedious operations, money-wasting, and slow. More importantly, there is no mature method to predict PMI accurately.

In the present study, a new experimental method, called lab-on-chip, is used to analyze protein and its degradation fragments, i.e., polypeptides. This method utilizes the Agilent 2,100 Bioanalyzer in combination with the protein LabChip kit, which simplifies the process of bioanalytical investigation and provides a system with standardized analysis handling and data processing (34). Although lab-on-chip cannot identify a polypeptide as a particular protein, the technology has been proven to be available for examining snake venom composition (35) and soybean cultivars in previous studies (36). It can perform molecular mass, migration time, peak height, peak area, relative concentration, and percentage of overall protein content and generate complete multi-peak spectrums of a sample. In addition, lab-on-chip is fast with minimal sample consumption, high throughput, and automatic quantitation (37), which means it is more appropriate for estimating PMI in practical work. At the same time, the abovementioned advantages also contribute to the united use of lab-on-chip and machine learning.

In the past decades, most studies have applied a single organ, such as the degradation of rat muscle proteins, used to estimate PMI by Zissler et al. (38). Although the two organs were used to estimate the time since death in the study by Mona Mohamed Abo El-Noor, the results of the heart and kidney were not analyzed jointly (39). In recent years, researchers from other fields have discovered that multi-organ fusion based on machine learning is more helpful to cancer diagnosis (40) and preclinical drugs than single organ (41). Hence, it is a beneficial trial that exploits multi-organ fusion and machine learning in estimating PMI. In the current study, lab-on-chip will analyze the polypeptide fragments in the lung, liver, kidney, and skeletal muscle of rat after death. We compare the performance of machine learning based on single and multiple organs to estimate the time since death and obtain the best prediction model based on multiple organs, which provides a new idea for forensic death time estimation.

## 2. Materials and methods

**FIGURE 1:**
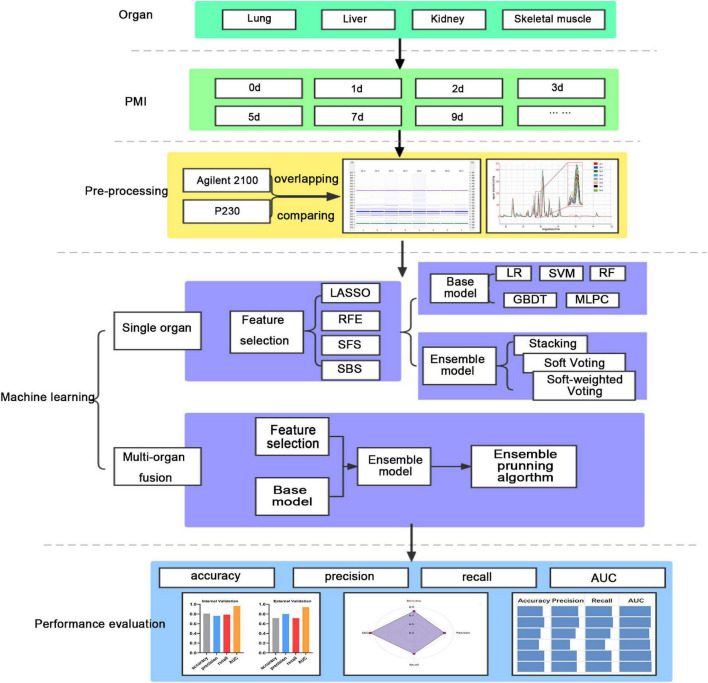
The workflow of this study.

This study’s workflow mainly involves the following (Figure 1). (1) Lab-on-chip analysis of the post-mortem degradation of polypeptides from the lung, liver, kidney, and skeletal muscle of rat at defined time points; (2) Base models (LR, SVM, RF, GBDT, and MLPC) and ensemble models (stacking, soft voting, and soft-weighted voting) evaluate the single organ’s performances to predict PMI; and (3) The ensemble model based on a multi-organ fusion strategy evaluates multi-organ performances to predict PMI.

### 2.1. Equipment, reagents, and supplies

A two-place balance (AX223ZH/E, OHAUS, China), vortex finder (VXMNFS, OHAUS, China), thermocell mixing block (MSC-100, Aosheng, China), heraeus sepatech (2-16PK, Sartorius, Germany), climate chamber (RX2-260B, Ningbo, China), and Agilent 2,100 Bioanalyzer (Agilent Technologies, Waldbronn, Germany) were used.

Deionized water for protein extraction, a Agilent Protein 230 LabChip^®^ kit (Agilent Technologies, CA, USA), and dithiothreitol (DTT, 1 M; Solarbio, Beijing, China) were used for the preparation of denaturant.

### 2.2. Animal sample

This study was approved by the Institutional Animal Care and Use Committee of Shanxi Medical University. Animals received humane care in conformity with the principles in the Guide for the Care and Use of Laboratory Animals protocol, published by the Ministry of the People’s Republic of China. This study was carried out in compliance with the ARRIVE guideline and evaluated and approved by the Institutional Animal Care and Use Committee of the Shanxi Medical University of China.

A total of 84 healthy male Sprague–Dawley rats, 10–12 weeks, weighing 200–230 g (provided by Animal Center of Shanxi Medical University) were housed in a cage with rat chow and water under a 12-h light–dark cycle at 22–25°C at a relative humidity of 40–60%. After 2 days, rats were sacrificed after pentobarbital anesthetization *via* cervical dislocation. The lung, liver, kidney, and right hind limb gastrocnemius muscle of each rat were harvested (200 mg ± 2 mg) at the fixed time points of 0, 1, 2, 3, 5, 7, 9, 12, 15, 18, 21, 24, 27, and 30 days (*n* = 6 rats) after sacrifice, and a total of 336 samples were placed in liquid nitrogen for quick freezing and stored at –80°C until analysis.

For external validation, 28 rats were taken according to the methods of the abovementioned experimental process. Each time point took two rats.

### 2.3. Water-soluble protein extraction and samples preparation

Analysis was performed according to the protocol provided by the manufacturer. A volume of 200 mg of the lung, liver, kidney, and skeletal muscle tissues were ground, added to deionized water containing 1% phenylmethylsulfonyl fluoride (PMSF) according to the ratio of 1:3.5 (w/v), then incubated on ice for 60 min, and centrifuged at 12,000 × *g* (15 min, 4°C). A volume of 4 μl solution per sample was diluted by mixing with 2 μl of the sample buffer with a reducing agent (DTT). The diluted solution and ladder (Agilent) were heated for 5 min at 95°C and then diluted with 84 μl H_2_O. Samples and ladder were loaded on the protein chip and measured immediately. To confirm the protein extraction process or the protein analysis process by lab-on-chip had avoided errors as much as possible, quality control samples were prepared.

### 2.4. Microfluidic LoaC electrophoresis

The protein profile of rat skeletal muscle using microfluidic capillary gel electrophoresis with laser-induced fluorescence (LIF) detection was carried out on the Bioanalyzer Agilent 2,100 using the Protein 230 Kit (Agilent Technologies, Waldbronn, Germany), which allows the separation of proteins from 14 to 230 kDa. According to the protocol, 4 μl of each tissue sample was mixed with 2 μl denaturing solution (35 mM dithiothreitol) in 0.5-ml tubes and denatured at 100°C for 5 min, incubated in ice for 2 min, and centrifuged for 15 s. Pure water was added to 100 μl, and samples were vortexed. Then, 6 μl of samples were added to each well of the chip. For the analysis, three biological replicates were used for each sample.

All reagents were provided with each LabChip kit, including the standard protein ladder containing different proteins with known concentrations and molecular weights that can be used for semi-quantitative analysis. The Agilent 2,100 Bioanalyzer separates and calculates the protein fragments based on the microfluidic capillary gel electrophoresis with LIF detection, where fluorescence intensities of proteins are measured. The migration times of polypeptide fragments were used to estimate the respective protein bands’ molecular weights, and the height was calculated to semi-quantify each protein fragment’s concentration. Data analysis performed with the Agilent 2,100 Expert software automatically determines molecular weight, concentration, and percentage of the sample’s total individual proteins.

### 2.5. Confirmation of polypeptide fragments and data preprocessing

All protein electrophoresis chromatography analyses were performed by “comparison” and “overlap” operations in the software to calibrate, identify, and adjust peaks according to the lower and upper markers. The same polypeptide fragments of each organ can be marked as the same number according to the molecular mass of these peptides, from minor to major. Numerical data such as protein molecular mass, peak height, and migration time are outputted for subsequent analysis.

It is essential to confirm the polypeptide fragments, which could be used as an indicator to estimate the PMI. The present study acquired the raw data through Agilent 2,100 Expert software, and all CSV data were imported into MS Excel. Then, the polypeptide fragments detected in five out of six biological replicate samples were identified as meaningful indicators for estimating PMI. The deviation of migration times less than 2% was considered the same polypeptide fragment in different samples.

Then, the datasets of each organ with 84 rats have been randomly divided into two, namely, the training dataset, which was made up of 70% of the dataset, and the testing dataset, also named internal validation, which comprised the remaining 30%, and standardized. For the external validation of 28 rats, the same data preprocessing was applied as mentioned earlier.

### 2.6. Machine learning

#### 2.6.1. Feature importance evaluated for machine learning

Feature selection, or feature ranking, reduces data processing time and memory requirements for machine-learning algorithms to deal with the essential predictors. In the present study, feature importance was evaluated through the least absolute shrinkage and selection operator (LASSO) (42), recursive feature elimination (RFE) (43), sequential forward selection (SFS), and sequential back selection (SBS) (44, 45).

#### 2.6.2. Sub-model training and evaluation for PMI using different organs

Five machine learning algorithms, including Logistic Regression (LR), Support Vector Machine (SVM), Random Forest (2), Gradient Boosting Decision Tree (GBDT), and Multilayer Perceptron Classier (MLPC), were implemented to predict PMI in the present study. The robustness and efficiency of 20 sub-models according to the four feature selection methods cross-match five machine learning algorithms are analyzed for each organ. The performance comparison analysis was performed by sequencing accuracy, precision, recall, and area under the ROC curve (AUC) of internal and external validation according to the order from good to wrong. And then, the ranking scores of all metrics were summed for each sub-model. Finally, the optimal classification model was determined by comparing the scores of 20 sub-models of each organ. It should be noted that the principle of this scoring method is to combine internal and external verification and comprehensive consideration of multiple evaluation indicators. Therefore, we believe that the model with the highest score has the highest comprehensive efficiency, which means that the model may not be the best in all indicators.

#### 2.6.3. Ensemble model development and evaluation for PMI based on single organ

Ensemble learning can improve the classifier’s performance by combining the trained sub-models contribution to solving the same classification problem in some studies (46). In the present study, there are three ensemble models, namely, stacking (47), soft voting (48), and soft-weighted voting (49), used to estimate the PMI based on the single organ. The accuracy, precision, recall, and AUC were calculated separately.

#### 2.6.4. Multi-organ fusion strategy and ensemble pruning algorithm

A framework that is suitable for multi-organ fusion analysis is proposed in this study. First, each organ’s best combinations of feature selection methods and sub-models were combined into a pipe. Four pipelines are used as four sub-models to complete each organ’s feature selection and PMI prediction. Then, four parallel pipelines were performed to predict PMI by the abovementioned three ensemble models. In this step, the four organs are fused to predict PMI. Finally, the ensemble models based on multi-organ fusion were compared with the optimal sub-models and ensemble models based on single organ.

After getting the best model, the ensemble pruning algorithm was applied to ensure the best combination of an organ. The ensemble pruning algorithm is a technique where the model starts with all possible members being considered and removes members from the ensemble until no further improvement is observed. This could be performed in a greedy manner where members are removed one at a time and only if their removal results in a lift in the performance of the overall ensemble.

## 3. Results

### 3.1. Characterization of polypeptide fragments after death

**FIGURE 2:**
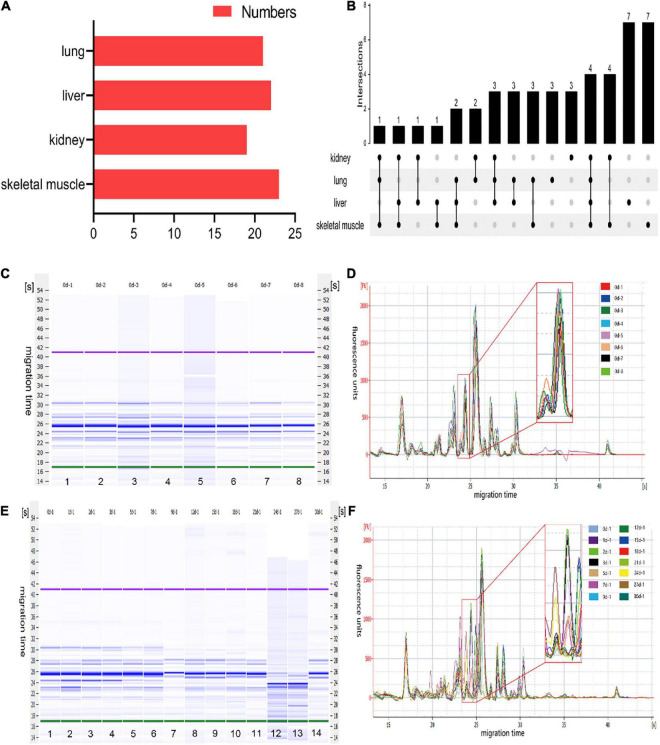
The characteristics of polypeptide fragments in different organs at different times after death. **(A)** The numbers of polypeptide fragments in different organs. **(B)** Co-expression analysis of polypeptide fragments in different organs. **(C)** The gel-like image of polypeptide fragments in skeletal muscle at the same time points after death. This figure is the simulated gel electrophoresis figure automatically given by Agilent 2,100 Bioanalyzer according to the molecular weight. Lanes 1–8 represent the gel diagram of eight skeletal muscle samples in 0 day after death. The migration time (s) is set on the side of the gel image. The purple bands at the top and the green bands at the bottom are the upper/lower ladder, which is the standard, respectively. The remaining blue bands are the detected protein fragments. The shade of the blue band represents the content of each protein fragment. **(D)** The electropherogram of skeletal muscle at the same time points after death in the microfluidic chip electrophoresis (LoaC) system. Multi-peak spectrums overlaid of different rats at the same time points after death, and there was no significant difference in peak height and number of peaks in the superposition of multi-peak spectra at the same time point after death of different rats. It is worth noting that there is a peptide peak around 24.5s in all samples at the same time point after death. **(E)** The gel-like image of polypeptide fragments in skeletal muscle at different time points after death. This figure is the simulated gel electrophoresis figure automatically given by Agilent 2,100 Bioanalyzer according to the molecular weight. Lanes 1–14 represent the gel diagram of 14 time points of skeletal muscle samples within 0–30 days after death. The migration time (s) is set on the side of the gel image. The purple bands at the top and the green bands at the bottom are the upper/lower ladder, which is the standard, respectively. The remaining blue bands are the detected protein fragments. The shade of the blue band represents the content of each protein fragment. **(F)** The electropherogram of skeletal muscle at different time points after death in the microfluidic chip electrophoresis (LoaC) system. The peak heights showed significant differences and a new peptide peak appears near 24.5 s by comparing multi-peak spectrums at different time points after death.

A total of 45 polypeptide fragments were identified with different migration times in the lung, liver, kidney, and skeletal muscle samples (Table 1). These polypeptide fragments may be highly correlated with the PMI, and 21, 22, 19, and 23 polypeptide fragments were found in the lung, liver, kidney, and skeletal muscle tissues, respectively (Figure 2A). Among these polypeptide fragments, 4 polypeptide fragments were detected in four organs, 7 polypeptide fragments were present in three organs, and 14 polypeptide fragments were present in two organs (Figure 2B). There were three polypeptide fragments specific to the kidney and lung but seven to the liver and skeletal muscle.

**TABLE 1:** The polypeptide fragments in the lung, liver, kidney, and skeletal muscle samples.

Polypeptide	Molecular mass (−X ± SD)	Migration time (−X ± SD)	Organs^a^
1	14.25 ± 0.45	20.68 ± 0.08	Lu^b^, Li^c^, K^d^, M^e^
2	15.53 ± 0.30	20.95 ± 0.05	M
3	17.61 ± 0.34	21.34 ± 0.06	M
4	19.65 ± 0.62	21.81 ± 0.13	K
5	25.58 ± 0.73	21.94 ± 0.15	Li
6	22.62 ± 0.83	22.36 ± 0.16	Li, K
7	23.84 ± 0.38	22.56 ± 0.07	M
8	24.54 ± 0.37	22.66 ± 0.06	Lu, Li
9	25.63 ± 0.69	22.93 ± 0.15	K, M
10	26.83 ± 0.35	23.12 ± 0.08	Lu, Li, M
11	29.43 ± 0.50	23.57 ± 0.14	Lu, K
12	31.68 ± 0.64	23.91 ± 0.10	K, M
13	32.92 ± 0.75	24.08 ± 0.12	Lu, Li, K
14	35.47 ± 0.91	24.40 ± 0.10	Lu, Li, M
15	39.65 ± 0.94	25.02 ± 0.18	Lu, Li, K, M
16	43.68 ± 0.82	25.59 ± 0.11	Lu, Li, K, M
17	45.15 ± 1.21	25.86 ± 0.17	Li
18	47.34 ± 0.91	26.19 ± 0.12	K
19	50.20 ± 1.11	26.46 ± 0.13	Lu, Li
20	51.97 ± 0.47	26.69 ± 0.09	K, M
21	53.06 ± 0.65	26.86 ± 0.08	Li
22	55.43 ± 0.43	27.15 ± 0.04	Li
23	57.44 ± 1.10	27.32 ± 0.16	Lu, K, M
24	58.43 ± 1.11	27.54 ± 0.18	Li, K, M
25	62.89 ± 0.70	28.08 ± 0.08	M
26	71.58 ± 1.57	28.80 ± 0.27	Lu, Li, K
27	74.83 ± 1.49	28.99 ± 0.16	Lu, M
28	78.23 ± 1.25	29.29 ± 0.07	M
29	82.13 ± 1.08	29.68 ± 0.09	Li
30	84.57 ± 0.77	29.88 ± 0.07	K
31	85.91 ± 0.98	29.90 ± 0.09	Lu, M
32	91.34 ± 1.04	30.30 ± 0.09	Lu
33	93.95 ± 1.91	30.59 ± 0.16	Lu, Li, K, M
34	104.86 ± 1.01	31.59 ± 0.08	Li
35	111.46 ± 5.41	32.09 ± 0.52	M
36	121.23 ± 1.82	32.98 ± 0.17	M
37	123.73 ± 2.40	33.23 ± 0.20	K, M
38	131.39 ± 1.01	33.90 ± 0.09	Li
39	135.25 ± 2.04	34.20 ± 0.17	Lu, Li
40	141.40 ± 1.35	34.71 ± 0.15	Lu, M
41	144.59 ± 2.03	35.04 ± 0.18	Li, M
42	154.94 ± 1.46	35.78 ± 0.11	Lu, K
43	179.02 ± 1.05	37.28 ± 0.07	Lu, Li, K
44	217.22 ± 0.98	39.60 ± 0.07	Lu
45	225.04 ± 3.44	40.08 ± 0.21	Lu

^a^Organs with polypeptide fragments.

^b^Lu represents lung.

^c^Li represents liver.

^d^K represents kidney.

^e^M represents skeletal muscle.

After further analysis of the data, we found that the content of the abovementioned polypeptide fragments was highly homogeneous in the samples with the same PMI (Figures 2C, D). The results showed no significant difference among the biological replicates, providing that the experimental operation was stable and reliable. In addition, the polypeptide fragments showed different peak heights at different PMIs (Figures 2E, F), which highly correlated with PMI.

**FIGURE 3:**
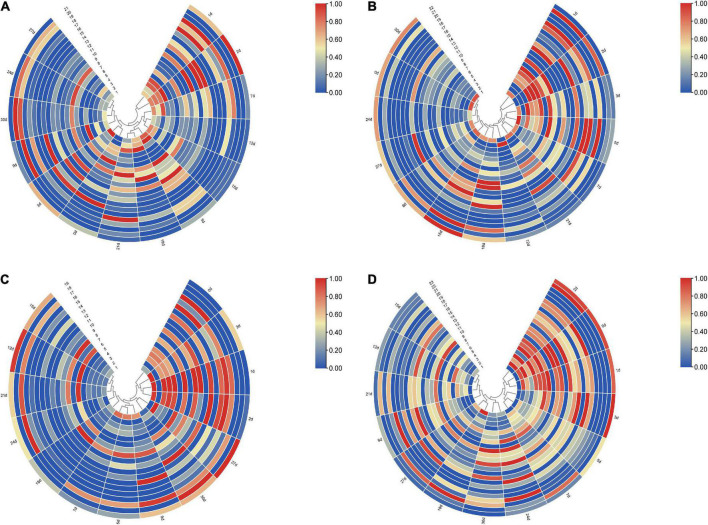
The clustering heat map based on the peak heights of polypeptide fragments in different organs. **(A)** Lung samples could be divided into five different stages, 0 and 3 days, 1 and 2 days, 5, 18, and 21 days, 7, 12, and 15 days, and 9, 24, 27, and 30 days were divided together, respectively. **(B)** Liver samples could be divided into five different stages, 1 and 2, 3, and 5 days, 7 and 21 days, 12 days, and 0, 9, 15, 18, 24, 27, and 30 days were divided together, respectively. **(C)** Kidney samples could be divided into four different stages, 0 to –3 days, 5, 7, and 9 days, 12, 15, 18, 21, and 24 days, and 27 and 30 days were divided together, respectively. **(D)** Skeletal muscle samples could be divided into five different stages, 0–2 days, 3, 5, and 7 days, 9, 12, 15, and 21 days, 18 and 27 days, and 24 and 30 days were divided together, respectively.

To further clarify the correlation between peptide fragment content and PMI, the earlier data were clustered using TB tools. It can be found from the clustering heat map that the death time of this experiment could be divided into five different stages according to the content of polypeptide fragments in the lung. Specifically, 0 and 3 days, 1 and 2 days, 5, 18, and 21 days, 7, 12, and 15 days, and 9, 24, 27, and 30 days were divided together (Figure 3A). Similarly, the samples can be distinguished into 5, 4, and 5 different periods according to the content of polypeptide fragments in the liver, kidney, and skeletal muscle (Figures 3B–D).

### 3.2. Performance of sub-models based on different organs

#### 3.2.1. Evaluating the sub-models by accuracy, precision, recall, and AUC

**FIGURE 4:**
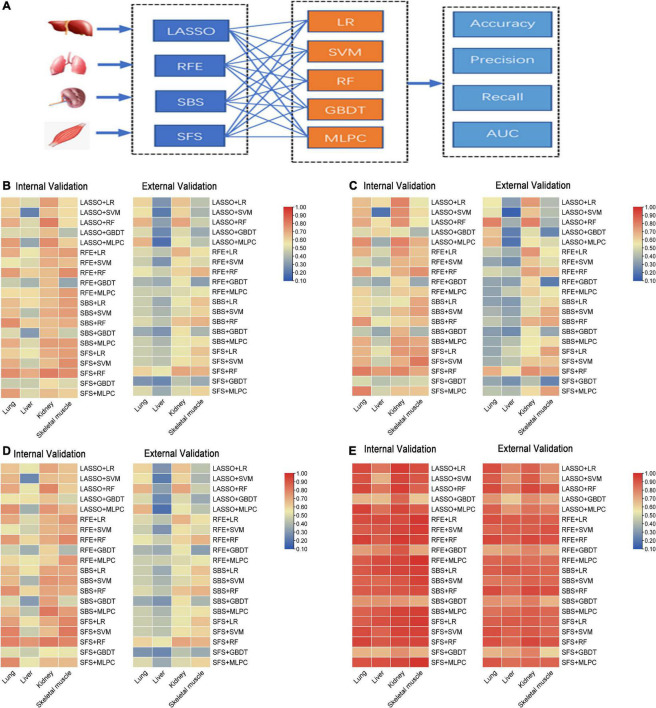
The performance of sub-models generated by cross-combination of four feature selection methods and five machine learning algorithms based on single organ. **(A)** Workflow of cross-combination of four feature selection methods and five machine learning algorithms to establish sub-models to predict PMI based on the lung, liver, kidney, and skeletal muscle. **(B)** The heat map on the left show accuracy of internal validation of sub-models based on lung, liver, kidney and skeletal muscle, and the heat map on the right shows the accuracy of external validation. **(C)** The heat map on the left show precision of internal validation of sub-models based on the lung, liver, kidney, and skeletal muscle, and the heat map on the right shows the precision of external validation. **(D)** The heat map on the left show recall of internal validation of sub-models based on the lung, liver, kidney, and skeletal muscle, and the heat map on the right shows the recall of external validation. **(E)** The heat map on the left shows AUC of internal validation of sub-models based on the lung, liver, kidney, and skeletal muscle, and the heat map on the right shows AUC of external validation.

To compare the predictive accuracy of four different organs in inferring the PMI, a total of 80 combined results were generated by cross-combining of four feature selection methods (e.g., LASSO, RFE, SBS, and SFS) and five machine learning algorithms (e.g., LR, SVM, RF, GBDT, and MLPC) (Figure 4A).

The accuracy, precision, recall, and AUC of sub-models with four organs are summarized in Figures 4B–E. As is shown in Figure 4B, the internal validation accuracy ranges of the lung, liver, kidney, and skeletal muscle were 0.462 (RFE + GBDT and SFS + GBDT)–0.769 (SBS + RF and SFS + RF), 0.231 (LASSO + SVM)–0.692 (SFS + RF), 0.577 (SFS + GBDT)–0.808 (LASSO + RF and SFS + RF), and 0.346 (RFE + GBDT)–0.769 (RFE + RF, SFS + SVM, and SFS + RF), respectively. Their external verification accuracies were 0.286 (SFS + GBDT)–0.679 (LASSO + RF and LASSO + MLPC), 0.179 (LASSO + MLPC)–0.536 (SFS + RF), 0.429 (SFS + GBDT)–0.714 (LASSO + RF and SFS + RF), and 0.321 (SFS + GBDT)–0.679 (RFE + RF, SBS + RF, and SFS + RF), respectively. Similarly, the analysis of Figures 4C–E shows that the model with the kidney as the detection sample performs best in precision, recall, and AUC evaluation indexes.

The abovementioned results indicated that the liver is the worst, and the kidney is the best to predict PMI among the four organs. As for the feature selection methods, the four feature selection methods cannot clearly distinguish the advantages and disadvantages. These results further show that LASSO, RFE, and SFS help determine feature subsets, which means that feature selection methods are necessary for different organs. It is particularly interesting that RF, the best machine learning algorithm in all organs, has advantages over other machine learning algorithms in predicting PMI, as mentioned earlier, while GBDT performed worst in the lung, kidney, and skeletal muscle. The four organs’ remaining indicators were similar results (Figures 4B–E).

#### 3.2.2. Screening optimal model by the ranking principle

The ranking scores principle described in the “Sub-models training and evaluation for PMI using different organs” section was used to compare the sub-models of each organ comprehensively. As is shown in Table 2, the best model combination in the lung and liver is SFS + RF, with scores of 146 and 149, respectively. The optimal sub-model of the kidney is LASSO + RF, which has a score of 149. The best sub-model of skeletal muscle is RFE + RF, which has a score of 139.

**TABLE 2:** The scores of sub-models generated by cross-combination of four feature selection methods and five machine learning algorithms.

Model	Lung	Liver	Kidney	Skeletal muscle
LASSO + LR	107.5	76	128	50.5
LASSO + SVM	100	15	99.5	47.5
LASSO + RF	135.5	83	149	59
LASSO + GBDT	59.5	57.5	27	19.5
LASSO + MLPC	137	29	93.5	64.5
RFE + LR	104.5	113.5	110	107
RFE + SVM	48	70.5	92.5	93.5
RFE + RF	132	135.5	112	139
RFE + GBDT	18.5	48.5	46	14
RFE + MLPC	53	125	51.5	96
SBS + LR	64.5	109.5	65	115.5
SBS + SVM	60.5	71	62	111.5
SBS + RF	118	119	93.5	116.5
SBS + GBDT	20.5	32.5	65.5	25.5
SBS + MLPC	69	87.5	102	104.5
SFS + LR	71	130.5	83	130.5
SFS + SVM	99.5	96	91.5	122.5
SFS + RF	146	149	143	134.5
SFS + GBDT	19.5	37	11	22
SFS + MLPC	116	94	54.5	106.5

**FIGURE 5:**
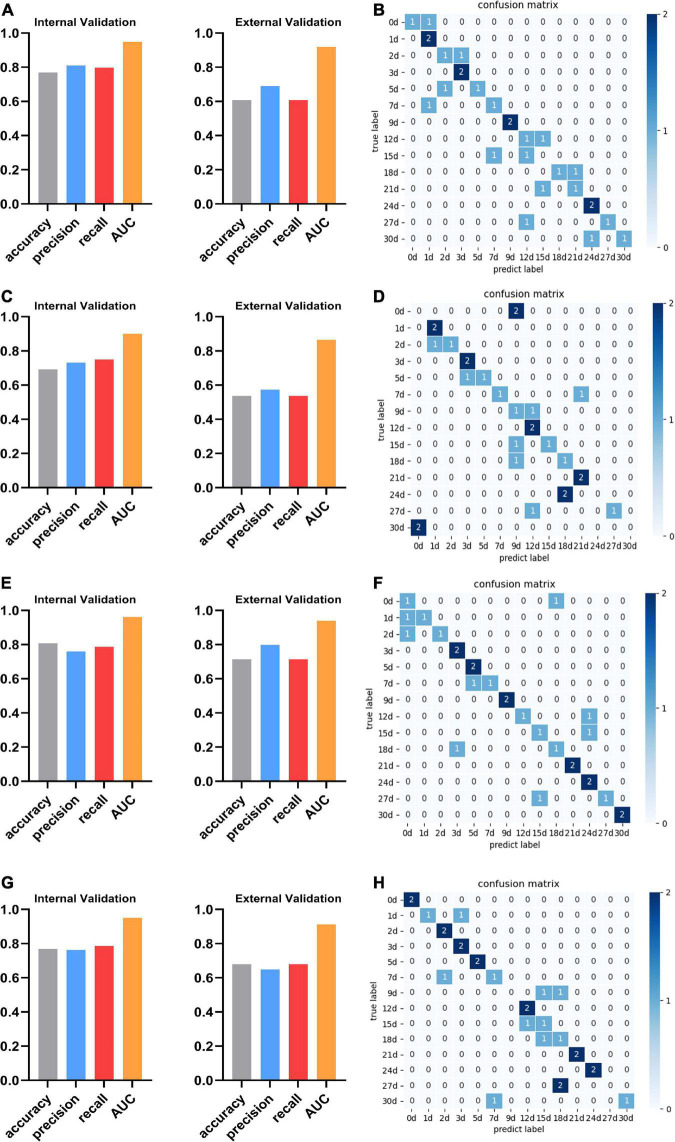
The performance and confusion matrix of the optimal sub-models with the lung, liver, kidney, and skeletal muscle. **(A)** The optimal sub-model of the lung is SFS + RF. The accuracy, precision, recall, and AUC of internal validation are 0.769, 0.810, 0.798, and 0.948, respectively. The accuracy, precision, recall, and AUC of external verification of this model are 0.607, 0.690, 0.607, and 0.919, respectively. **(B)** The confusion matrix of SFS + RF for the lung shows that the external validation samples were completely predicted correctly only at 1, 3, 9, and 24 days. The external validation predictions were wrong at 15 days after death. There was a misjudgment in the samples at other PMI. **(C)** The optimal sub-model of the liver is SFS + RF. The accuracy, precision, recall, and AUC are 0.692, 0.732, 0.750, and 0.900, respectively. The accuracy, precision, recall, and AUC of external verification are 0.536, 0.574, 0.536, and 0.865, respectively. **(D)** The confusion matrix of SFS + RF for the liver shows that the external validation samples of Liver were completely predicted correctly at 1, 3, 12, and 21 days. The external validation predictions were wrong at 0, 24, and 30 days after death. There was a misjudgment in the samples at other PMI. **(E)** The optimal sub-model of the kidney is LASSO + RF. The accuracy, precision, recall, and AUC are 0.808, 0.760, 0.786, and 0.962, respectively. The accuracy, precision, recall, and AUC of external verification are 0.714, 0.798, 0.714, and 0.939, respectively. **(F)** The confusion matrix of LASSO + RF for the kidney shows that the external validation samples of Kidney were completely predicted correctly at 3, 5, 9, 21, 24, and 30 days, and there was a misjudgment in the samples at other PMI. **(G)** The optimal sub-model of skeletal muscle is RFE + RF. The accuracy, precision, recall, and AUC are 0.769, 0.762, 0.786, and 0.951, respectively. The accuracy, precision, recall, and AUC of external verification of this model are 0.679, 0.649, 0.679, and 0.912, respectively. **(H)** The confusion matrix of RFE + RF for skeletal muscle shows that the external validation samples of skeletal muscle were completely predicted correctly at 0, 2, 3, 5, 12, 21, and 24 days, The external validation predictions were wrong at 9 and 27 days after death. There was a misjudgment in the samples at other PMI.

We found that the kidney is more suitable than other organs to predict PMI, comparing the performance of the best models for each organ. In optimal sub-models of four organs, 0.808 and 0.714 are the highest internal and external validation accuracies based on LASSO-RF of the kidney (Figure 5E), respectively. In Figure 5F, the confusion matrix of external verification of the kidney showed that eight samples were misjudged, and many miscalculations in the prediction results of the kidney were found at 0–2 days and 12–18 days after death. Next, the internal validation of the lung and skeletal muscle is 0.769 based on SFS-RF. The former’s accuracy of external validation is 0.607 lower than the latter, which is 0.679 (Figures 5A, G). The liver is the worst organ to predict PMI; the accuracy is 0.692 and 0.536 in internal and external validation using SFS-RF, which is the best classification model for the liver (Figure 5C). As shown in Figures 5B, D, H, there are 11, 13, and 9 samples of the lung, liver, and skeletal muscle, respectively, which were wrongly judged in their external verification.

### 3.3. Performance of the single organ based on ensemble models

Considering that different prediction models have different prediction performances in four organs, this experiment will cross-combine the four feature selection methods and three ensemble models mentioned earlier to establish an ensemble model to improve the performance of PMI estimation in a single organ.

**FIGURE 6:**
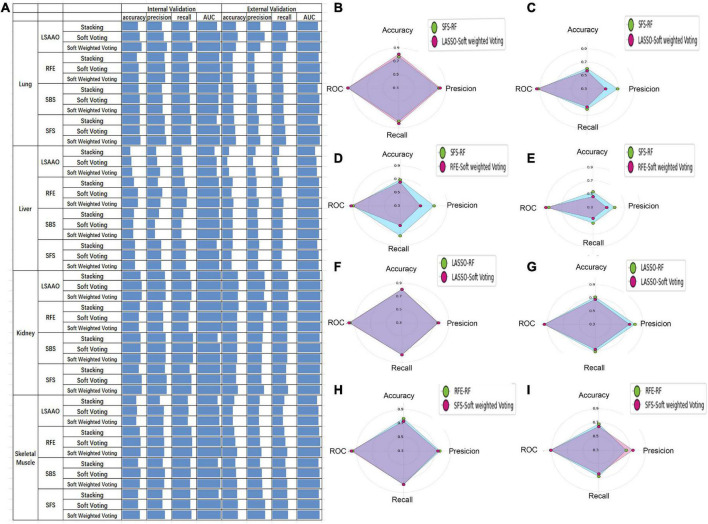
The prediction performance of ensemble model and best sub-model based on single organ. **(A)** Histogram performance of internal and external validation of four organs cross-combining of four feature selection methods and three ensemble models. **(B)** Radar map of SFS-RF and LSAAO-soft-weighted voting based on internal validation of lung. **(C)** Radar map of SFS-RF and LSAAO-soft-weighted voting based on external validation of lung. **(D)** Radar map of SFS-RF and RFE-soft-weighted voting based on internal validation of liver. **(E)** Radar map of SFS-RF and RFE-soft-weighted voting based on external validation of liver. **(F)** Radar map of LSAAO-RF and LSAAO-soft voting based on internal validation of kidney. **(G)** Radar map of LSAAO-RF and LSAAO-soft voting based on external validation of kidney. **(H)** Radar map of RFE-RF and SFS-soft-weighted voting based on internal validation of skeletal muscle. **(I)** Radar map of RFE-RF and SFS-soft-weighted voting based on external validation of skeletal muscle.

The performance of the ensemble models of four organs is shown in Figure 6A. In the validation of the lung, LASSO + soft-weighted voting generated the highest accuracy of 0.808 in the internal validation, while LASSO + soft voting generated the highest accuracy of 0.643 in the external validation. The best accuracy of internal validation based on the liver is 0.654, which was obtained by RFE + soft voting and RFE + soft-weighted voting. The accuracy of RFE + soft-weighted voting for external validation of the liver had reached 0.464. For kidneys, the accuracy for internal validation of LASSO + soft voting, LASSO + soft-weighted voting, SFS + soft voting, and SFS + soft-weighted voting was 0.808, while the optimal accuracy for external validation of LASSO + soft voting and RFE + stacking was 0.679. The highest accuracy for internal validation of skeletal muscle was 0.769, and the combined strategies were RFE + soft voting and RFE + soft-weighted voting, respectively. Furthermore, the external validation accuracy of SFS + soft voting and SFS + soft-weighted voting for skeletal muscle is 0.643. The details of the precision, recall, and AUC have similar results, as shown in Figure 6A.

According to the ranking principle described in the “Sub-models training and evaluation for PMI using different organs” section, the optimal ensemble model of each organ was screened in this experiment. The best ensemble model in the lung is LASSO + soft-weighted voting with ranking scores of 89, and the internal and external validation accuracies were 0.808 and 0.571, respectively (Table 3). Specifically, the optimal ensemble model of RFE + soft-weighted voting based on the liver was 89.5, and the internal and external validation accuracies were 0.654 and 0.464, respectively. The internal and external verification accuracies for the kidney are 0.808 and 0.679, respectively, based on LASSO + soft voting, which has the highest score of 76. The best ensemble model of skeletal muscle is SFS + soft-weighted voting, which scored 81, and the internal and external accuracy were 0.731 and 0.643, respectively.

**TABLE 3:** The scores of ensemble models based on four organs.

Model	Lung	Liver	Kidney	Skeletal muscle
LASSO + stacking	39.5	15.5	54.5	17
LASSO + soft voting	78.5	17	76	26
LASSO + soft weighted voting	89	30	71.5	21.5
RFE + stacking	24	56.5	60.5	45.5
RFE + soft voting	38.5	70	46	69.5
RFE + soft weighted voting	41	89.5	45	80.5
SBS + stacking	28	52	41.5	36.5
SBS + soft voting	42.5	51	49.5	68.5
SBS + soft weighted voting	45.5	54.5	49.5	67
SFS + stacking	50.5	65	23	46.5
SFS + soft voting	64	61.5	41.5	64.5
SFS + soft weighted voting	83	61.5	65.5	81

In the present study, each organ’s ensemble model was compared with the best sub-model of the same organ to determine whether the integrated model can improve the PMI prediction performance. Compared with SFS-RF, the best sub-model of the lung, although all metrics of internal validation are slightly improved, its external validation metrics significantly decreased according to LASSO + soft-weighted voting (Figures 6B, C). The RFE + soft-weighted voting model based on the liver predicts PMI with the most indicators lower than the best sub-model except for the AUC of internal and external validation (Figures 6D, E). Compared with the optimal kidney sub-model, the LASSO + soft voting model weakly improves the precision and AUC of internal validation (Figures 6F, G). By comparing SFS-soft-weighted voting with SFS-RF of skeletal muscle, the former only has feeble improvement in AUC of internal validation and precision of external validation (Figures 6H, I).

The abovementioned results indicated that the SFS + RF was the optimal model for predicting PMI based on the kidney. However, the single organ ensemble model could not effectively improve the PMI prediction performance. Therefore, in the multi-organ fusion based on ensemble model construction, the optimal sub-model performance will be compared with other models’ performance in predicting PMI.

### 3.4. Performance of multi-organ fusion based on ensemble models

**FIGURE 7:**
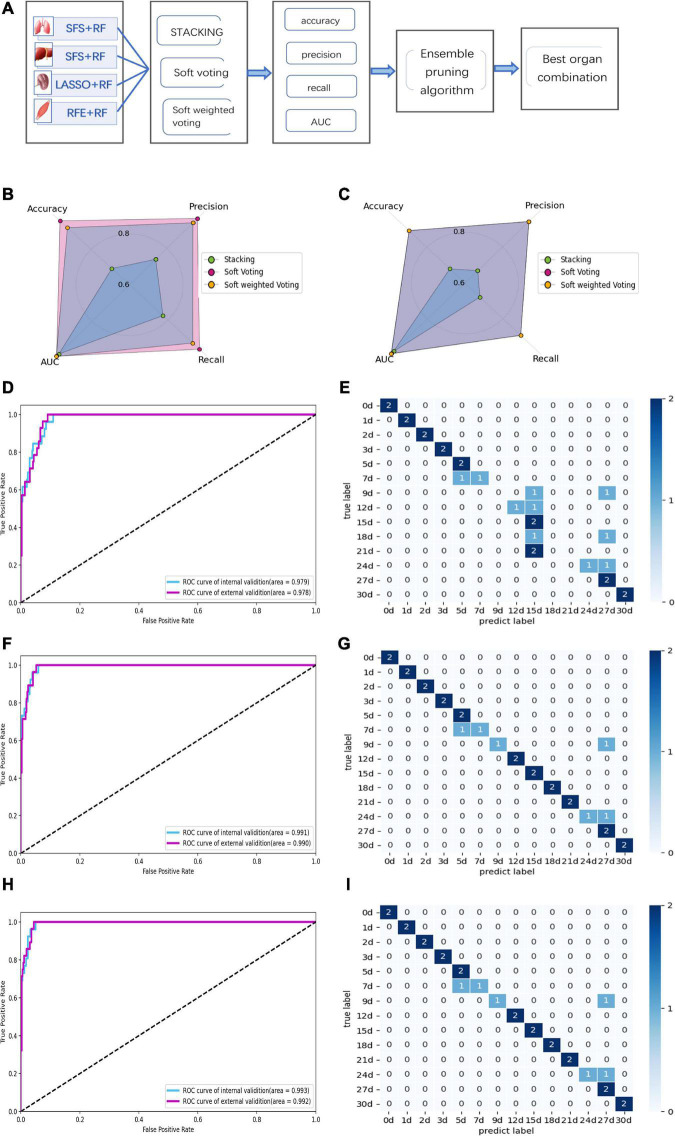
Performance of multi-organ fusion strategy to predict PMI. **(A)** Framework of multi-organ fusion strategy to predict PMI. **(B)** Accuracy, precision, recall, and AUC of internal validation for stacking are 0.692, 0.740, 0.774, and 0.979, respectively. Accuracy, precision, recall, and AUC of internal validation for soft voting are 0.962, 0.964, 0.964, and 0.991, respectively. Accuracy, precision, recall, and AUC of internal validation for soft-weighted voting are 0.923, 0.94, 0.929, and 0.993, respectively. **(C)** Accuracy, precision, recall, and AUC of external validation for stacking are 0.679, 0.668, 0.679, and 0.978, respectively. Accuracy, precision, recall and AUC of external validation for soft voting are 0.893, 0.94, 0.893, and 0.99, respectively. Accuracy, precision, recall, and AUC of external validation for soft-weighted voting are 0.893, 0.94, 0.893, and 0.992, respectively. **(D)** The ROC curve of internal and external validation for the stacking model based on multi-organ fusion strategy. **(E)** The confusion matrix of external validation for the stacking model, the mispredictions occurred 7 to 12 days and 18 to 24 days after death. **(F)** The ROC curve of internal and external validation for the soft voting model based on multi-organ fusion strategy. **(G)** The confusion matrix of external validation for the soft voting model. The external validation samples were predicted incorrectly at 7, 9, and 24 days of PMI. **(H)** The ROC curve of internal and external validation for the soft-weighted voting model based on multi-organ fusion strategy. **(I)** The confusion matrix of external validation for the soft-weighted voting model. The samples were mispredicted at 7, 9, and 24 days.

Since the single-organ ensemble strategy cannot improve the prediction efficiency of PMI, we further focus on the multi-organ integration strategy. Figure 7A shows the appropriate multi-organ fusion model establishment steps for estimating PMI. In brief, the best combinations of feature selection methods and sub-models in the lung, liver, kidney, and skeletal muscle were piped based on a multi-organ fusion strategy. Then, the ensemble model with the highest scores was selected by comparison. Finally, the ensemble pruning algorithm integrates multi-organ data based on the optimal model for PMI estimation.

By comparing the multi-organ integration model’s internal and external verification accuracies, the soft voting fusion strategy has an absolute advantage with the internal and external verification accuracies of 0.962 and 0.893, respectively. In contrast, the staking model had the worst performance, and its internal and external validation accuracy is even lower than the single-organ optimal model based on the kidney, with only 0.692 and 0.679. The performance of soft-weighted voting was similar to that of soft voting, with internal and external validation accuracies of 0.923 and 0.893 (Figures 7B, C).

Although the AUC values of the internal and external validation of the three fusion strategies are all higher than 0.97, the confusion matrix results show that some samples are still misjudged according to the external validation (Figures 7D–I). The sample prediction error is mainly more than 15 days after death, indicating that if the prediction results show that the PMI exceeds 15 days, the prediction accuracy decreases and the credibility decreases.

The ensemble pruning algorithm showed that the optimal combination of multiple organs was four organs, i.e., lung, liver, kidney, and skeletal muscle, used in the present study to infer the PMI. Furthermore, soft voting and soft-weighted voting can significantly improve the prediction performance of PMI based on the multi-organ fusion strategy (Table 4).

**TABLE 4:** The summary of all the optimal models is based on single organ sub-models, single organ ensemble models, and multi-organ fusion strategy.

Organ	Best model	Internal validation				External validation			
		ACC^a^	PRE^b^	REC^c^	AUC	ACC	PRE	REC	AUC
Lung	SFS + RF	0.769	0.81	0.798	0.948	0.607	0.690	0.607	0.919
	LASSO + soft weighted voting	0.808	0.827	0.833	0.949	0.571	0.536	0.571	0.941
Liver	SFS + RF	0.692	0.732	0.75	0.9	0.536	0.574	0.536	0.865
	RFE + soft weighted voting	0.654	0.56	0.595	0.924	0.464	0.474	0.464	0.901
Kidney	LASSO + RF	0.808	0.76	0.786	0.962	0.714	0.798	0.714	0.939
	LASSO + soft weighted voting	0.808	0.767	0.786	0.974	0.643	0.683	0.643	0.94
Skeletal muscle	RFE + RF	0.769	0.762	0.786	0.951	0.679	0.649	0.679	0.912
	SFS + soft weighted voting	0.731	0.738	0.786	0.966	0.643	0.735	0.643	0.91
Multi-organ fusion	Stacking	0.692	0.74	0.774	0.979	0.679	0.668	0.679	0.978
	Soft voting	0.962	0.964	0.964	0.991	0.893	0.94	0.893	0.99
	Soft weighted voting	0.923	0.94	0.929	0.993	0.893	0.94	0.893	0.992

^a^ACC represents accuracy.

^b^PRE represents precision.

^c^REC represents recall.

### 3.5. Comparison of lab-on-chip and traditional protein detection methods

To further clarify the superiority of the analysis method and its application value in forensic practice, we summarize the main improvements of the proposed approach compared to the traditional methods. And the terms include whether the required instruments are expensive, whether the detection methods are cumbersome, and the length of analysis time. The results show that the present study’s detection method and analysis strategy have good application prospects for estimating PMI (Table 5).

**TABLE 5:** Comparison of lab-on-chip and traditional protein detection methods.

	Lab-on-chip	Traditional methods		
		Western-blotting	ELISA	Protein mass spectrometry
Operations	Simplify	Complex	Complex	Complex
Sample consumption	Minimal	Major	Major	Minimal
Expenditure	Cheap	Cheap	Cheap	Expensive
Speed	Less than 30 min	Slow	Fast	Slow
Equipment	Only 2,100 Bioanalyzer	Variety	Few	Variety
Identify particular protein	No	Yes	Yes	Yes
Quantitation	Automatic	Semiquantitative	Semiquantitative	Automatic
High throughput	Yes	No	No	Yes
Data processing	Use machine learning	Manual analysis	Manual analysis	Use machine learning
Predict performance	Excellent	Poor	Poor	Good
Witnessed inspections	Yes	No	Yes	No

## 4. Discussion

Protein is one of the important components of an organism, so forensic pathologists have always used the analysis of protein degradation after death as an auspicious tool to determine PMI. Previous studies have shown that some specific proteins and their degradation products (e.g., desmin, cTnT, and calpain 1) could be used as markers for specific time intervals of post-mortem decomposition (50). In contrast, many protein detection methods have tested their applicability for predicting PMI. However, these technologies are complex, time-consuming, and expensive, but more importantly, the accuracy is not enough to infer PMI (51).

In the present study, the lab-on-chip combines Agilent 2,100 biological analyzer and the Protein 230 Plus LabChip kits, enabling the separation of polypeptides in the 14–230 kDa range. This technique allows the analysis of 10 samples in 30 min and avoids all the cumbersome post-electrophoresis procedures required for SDS-PAGE analysis, including staining, destaining, and storage, and does not need additional image analysis equipment. It is worth noting that the technology can directly display the results as gel-like images and electrophoretograms. It also can output the characteristics of each polypeptide peak as numerical data, such as molecular mass, peak height, and migration time. More importantly, the technology can simultaneously analyze multiple polypeptides or their degradation fragments of a sample. With this high-throughput advantage, this technology will help establish a human sample database and then realize the prediction of human samples with unknown PMI in the future.

The results of this study showed that the prediction accuracy of the kidney was the highest, followed by the lung and skeletal muscle, and that of the liver was the lowest when applying sub-models based on a single organ to predict PMI. The reason may be that the kidney, as a deep organ in the organism, is less affected by the outside world and less protease. The lung and skeletal muscles are greatly affected by the external environment because of gas exchange and relative superficial organs. The result of the liver was the lowest mainly because the detoxification organ of the organism contains many proteolytic enzymes.

According to the results mentioned in the “Performance of the single organ based on ensemble models” section, we found that the performance of ensemble models based on single organ is worse than that of the sub-model. When generating ensemble models, some fundamental principles should be considered. The first is diversity, which means the machine learning algorithm participating in ensemble learning should have enough diversity to obtain ideal prediction performance. The second is prediction performance, which means the individual machine learning algorithm should be as high as possible (52, 53). In the present research, we have used multiple models to ensure the diversity of algorithms. However, the disadvantage is that we have not deleted the worst-performing sub-models, such as GBDT, which may lead to the low accuracy of the integrated model.

In the current study, we designed a multi-organ fusion strategy combining multiple organs to predict PMI. The soft-voting and soft-weighted voting model based on multi-organ fusion strategy improved the predictive performance of internal and external verification. The results show that the soft-voting model drastically improved the accuracy of internal verification from 0.808 to 0.962 and the accuracy of external verification from 0.714 to 0.893. The reason may be that the essence of a multi-organ fusion strategy is to fuse and analyze multiple training datasets to fit different base models. It helps to integrate the characteristics of different organs better and increases the amount of data (53). Another possible reason is that we choose the optimal sub-model of the four organs in the multi-organ fusion strategy to have enough diversity to obtain ideal prediction performance (54).

Through this study, we also found significant differences in the predictive power of different ensemble models, which means it is necessary to compare and screen them. Compared with the Lu et al.’s study, they used the same four organs combined with mass spectrometry and multi-organ fusion to predict PMI, with an accuracy of 0.93 based on a stacking ensemble (55). However, the performance of the stacking ensemble was not satisfactory in our research. On the contrary, the accuracy of soft voting reached 0.96, which may be related to the different analytical techniques.

Ensemble pruning methods, called ensemble selection methods, aim to reduce ensemble models’ complexity. These methods search for a subset of ensemble members that performs to some extent as well as the original ensemble (56). This method can reduce the size of the ensemble model, save training time, and improve accuracy and robustness (57). Hence, in our study, we also used the ensemble pruning algorithm to select the optimal subsets of base models for multi-organ fusion, which also means that we can determine the optimal multi-organ combination for the estimation of PMI. Finally, we obtained that the optimal organ combination is the lung, liver, kidney, and skeletal muscle for predicting time since death. This result after pruning also suggests that we should try to use more organs to find the best organ combination to infer future PMI.

In forensic medicine, estimating the PMI is influenced by many internal and external factors such as temperature and humidity, body weight, and disease. The limitations should be avoided in future studies, such as considering more influencing factors and increasing the number of human samples. Although the current experiment involves an idealized condition, we have proven a new analysis method, lab-on-chip combined with a machine learning algorithm, could use to predict the PMI. Furthermore, the multi-organ fusion strategy can significantly improve the performance of PMI prediction.

## Data availability statement

The original contributions presented in this study are included in the article/supplementary material, further inquiries can be directed to the corresponding author.

## Ethics statement

This animal study was reviewed and approved by the Institutional Animal Care and Use Committee of Shanxi Medical University.

## Author contributions

Q-XD, J-HS, and SZ conceived the idea and drafted the manuscript. F-HL, X-JL, and LW established animal model and lab-on-chip experiment. JC and J-HS conceived the study and performed some interpretation of the results. Q-QJ and KR performed data collection and mathematical model construction. JZ and PH interpreted the results and modify the manuscript. All authors read and approved the final version of the manuscript.
